# 3-O-acetylrubiarbonol B preferentially targets EGFR and MET over rubiarbonol B to inhibit NSCLC cell growth

**DOI:** 10.1371/journal.pone.0329706

**Published:** 2025-09-08

**Authors:** A-Young Nam, Sang Hoon Joo, Na Yeong Lee, Goo Yoon, Jin Woo Park, MinKyun Na, Jung-Hyun Shim

**Affiliations:** 1 Department of Biomedicine, Health and Life Convergence Sciences, BK21 Four, College of Pharmacy, Mokpo National University, Muan, Republic of Korea; 2 College of Pharmacy, Daegu Catholic University, Gyeongsan, Republic of Korea; 3 Department of Pharmacy, College of Pharmacy, Mokpo National University, Muan, Republic of Korea; 4 College of Pharmacy, Chungnam National University, Daejeon, Republic of Korea; 5 The China-US (Henan) Hormel Cancer Institute, Zhengzhou, People’s Republic of China; IHRC, Inc. (Human Resource Service Administration), UNITED STATES OF AMERICA

## Abstract

Non-small cell lung cancer (NSCLC) is one of the leading causes of cancer-related deaths, remaining a significant challenge in terms of early detection, effective treatment, and improving patient survival rates. In this study, we investigated the anticancer mechanism of rubiarbonol B (Ru-B) and its derivative 3-*O*-acetylrubiarbonol B (ARu-B), a pentacyclic terpenoid in gefitinib (GEF)-sensitive and -resistant NSCLC HCC827 cells. Concentration- and time-dependent cytotoxicity was observed for both Ru-B and ARu-B. The *in vitro* kinase assay showed that ARu-B treatment inhibited epidermal growth factor receptor (EGFR), mesenchymal-epithelial transition (MET), and AKT1, and their phosphorylation in HCC827 cells. A molecular docking model suggested that ARu-B could interact with EGFR and MET in different ways, either by binding to the ATP pocket or the substrate pocket. ARu-B induced reactive oxygen species (ROS) generation and cell cycle arrest. The induction of apoptosis through caspase activation was confirmed by preventing cytotoxicity with Z-VAD-FMK pretreatment. Taken together, ARu-B inhibited the growth of both GEF-sensitive and GEF-resistant NSCLC cells by targeting EGFR, MET, and AKT and inducing ROS generation and caspase activation. Further studies on ARu-B can improve the treatment of chemotherapy-resistant NSCLC through the development of effective ARu-B-based anticancer agents.

## Introduction

While the incidence rate and mortality of lung cancer have been declining over the last three decades, more than two million lives are lost due to lung cancer every year, and lung cancer remains the leading cause of cancer-related deaths [1]. Non-small cell lung cancer (NSCLC) is a common type of lung cancer with a high mortality risk [2]. Multiple factors may contribute to the occurrence of NSCLC, which include mutations in receptor tyrosine kinases, such as EGFR, MET, and ALK and the Ras family oncogene KRAS [3]. Mutations in EGFR, MET, and KRAS individually drive oncogenesis through aberrant signaling pathways; however, as they accumulate, this growth becomes uncontrollable. Targeted therapies have been developed to inhibit aberrant signaling pathways, aiming to block uncontrolled cell proliferation, reduce tumor growth, and counteract mutations in various receptor tyrosine kinases [4]. Unlike traditional chemotherapy, these target-specific anticancer drugs are meant to effectively block the growth of cancer cells while causing little, if any, harm to non-cancer cells. However, resistance can develop to targeted therapies, and many studies have reported acquired resistance to specific tyrosine kinase inhibitors (TKIs), such as gefitinib (GEF) [5].

The human non-small cell lung cancer (NSCLC) cell line HCC827, harboring a mutation in EGFR, is sensitive to EGFR TKIs such as GEF and erlotinib [6]. However, exposure to GEF leads to the development of resistance, and GEF-resistant HCC827 (HCC827GR) cells have MET amplification. This resistance could be dealt with by targeting both GEF and MET simultaneously [7]. Additionally, modulating reactive oxygen species (ROS) may induce apoptosis in cancer cells, further intensifying the stress on cells treated with GEF and MET inhibitors [8]. The regulation of ROS at the cellular level is important in cell physiology and pathophysiology, and ROS induction and EGFR signaling are interrelated.

**Fig 1:**
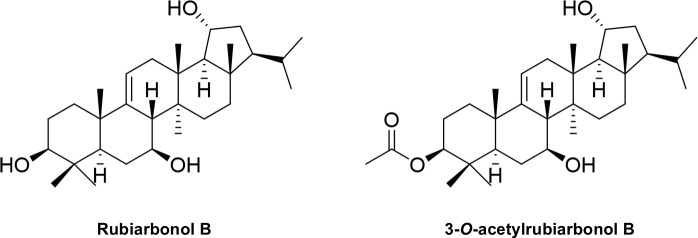
Chemical structure of rubiarbonol B (Ru-B) and 3-*O*-acetylrubiarbonol B (ARu-B).

Rubiarbonol B (Ru-B; Fig 1 left) is a pentacyclic triterpenoid isolated from *Rubia philippinensis* [9]. Several compounds in this class exert antitumor activity by modulating various signaling pathways regulating apoptosis, angiogenesis, and cell cycle progression [10]. The induction of apoptosis and cell cycle arrest by rubiarbonol G and myrotheols A has been reported [11,12]. Recently, we reported that Ru-B caused the apoptosis of RIPK3-expressing colorectal cancer cells [13]. Ru-B caused the formation of a ripoptosome to induce apoptosis and necroptosis. In this study, we investigated the antitumor activity of Ru-B and 3-*O*-acetylrubiarbonol (ARu-B; Fig 1 right), an acetylated form of Ru-B. The antiproliferative effect of Ru-B and ARu-B was examined in HCC827 and HCC827GR cells. The kinase activity of EGFR, MET, and AKT was measured in the presence of Ru-B or ARu-B to determine if Ru-B or ARu-B functioned as an inhibitor.

## Materials and methods

### Chemicals

Rubiarbonol B (purity > 95%, Fig 1 and S1 Fig) was obtained from the previous study on the roots of *Rubia philippinensis* [9]. ARu-B was isolated from the roots of *Rubia philippinensis* [14], which was prepared for the biological evaluation in this study (purity > 95%, Fig 1). ARu-B: White amorphous powder; [α] + 22 (*c* 0.1, CHCl_3_); UV (MeOH) λ_max_ (log ɛ) 243 (4.25); IR (CHCl_3_) *ν*_max_ 3330, 2958, 1734, 1376, 1049 cm^−1^; HRESIMS *m*/*z* 523.3756 [M + Na]^+^ (calcd for C_32_H_52_O_4_Na, 523.3763); ^1^H and ^13^C NMR data (S1 Table).

### Reagent and antibodies

Basal Medium Eagle (BME) and N-Acetyl-L-cysteine (NAC) were obtained from Sigma-Aldrich (St. Louis, MO, USA). 0.25% trypsin-EDTA solution, Fetal bovine serum (FBS), Phosphate Buffered saline (PBS), and Penicillin-Streptomycin were purchased from Gibco (Carlsbad, CA, USA). GEF (184475-35-2) was procured from Cayman Chemical (Ann Arbor, MI, USA). AZD5363 (Capivasertib, S8019) and Savolitinib (SAV, S7674) were obtained from Selleck Chemicals (Houston, TX, USA). Dulbecco’s Modified Eagle Medium (DMEM) and Roswell Park Memorial Institute (RPMI) medium and were purchased from Welgene (Gyeongsan-si, Gyeongsangbuk-do, KR). Z-VAD-FMK obtained from Enzo Biochem (Nassau, NY, USA) and Thiazolyl blue tetrazolium bromide (MTT) purchased from Biosesang (Yongin-si, Gyeonggi-do, KR). Primary antibodies to β-actin (Mouse, 1:1000, sc-47778, AB_626632), cyclin D1 (Mouse, 1:1000, sc-20044, AB_627346), CDK4 (Mouse, 1:1000, sc-70831, AB_1121416), CDK6 (Mouse, 1:1000, sc-7961, AB_627242), p27 (Mouse, 1:1000, sc-56338, AB_785027), caspase3 (Rabbit, 1:1000, sc-7148, AB_637828), EGFR (Mouse, 1:1000, sc-71033, AB_1122467), Bcl-2 (Mouse, 1:500, sc-7382, AB_626736), Mcl-1 (Mouse, 1:1000, sc-12756, AB_627915), and Bad (Mouse, 1:1000, sc-8044, AB_626717) were obtained from Santa Cruz Biotechnology (Dallas, TX, USA), while poly ADP-ribose polymerase (Rabbit, 1:1000, 9542S, AB_2160739), phosphorylated (p)-AKT (Rabbit, 1:1000, 4060S, AB_2315049), p-EGFR (Rabbit, 1:1000, 3777S, AB_2096270), p-MET (Rabbit, 1:1000, 3077S, AB_2143884), AKT (Rabbit, 1:1000, 4691S, AB_915783), MET (Rabbit, 1:1000, 8198S, AB_10858224), and Bax (Rabbit, 1:1000, 5023S, AB_10557411) were procured from Cell Signaling Technology (Danvers, MA, USA).

### Cell lines and treatment

HCC827 (GEF-sensitive NSCLC cell line) and HEKa (Human epidermal keratinocytes) cells were purchased from the ATCC (American Type Culture Collection, Manassas, USA). HCC827GR (GEF-resistance HCC827 NSCLC cell line) cells were kindly provided by Professor Pasi A. Jänne (Department of Medical Oncology, Dana-Farber Cancer Institute, Boston, MA, USA) and were maintained in 0.1 μM GEF [15]. HCC827 and HCC827GR cells were seeded in RPMI containing with 10% FBS and 100 U/mL Penicillin-Streptomycin (P/S). HEKa cells were seeded in DMEM (10% FBS and 100 U/mL P/S). The cells treated with ARu-B, Ru-B, GEF, or SAV for 24 h or 48 h. The NSCLC cells were pre-treated with ROS scavenger (NAC, 4 mM) or pan-caspase inhibitor (Z-VAD-FMK, 12 μM) for 3 h.

### Cell proliferation analysis

Cell proliferation determined by the MTT assay. After being treated for 24 or 48 h, the cells were added 1 mM of MTT reagent and incubated at 37°C. Production of each well was resolved in DMSO and detected at 570 nm by a microplate reader (Thermo Fisher Scientific, Waltham, USA). IC_50_ values were calculated by linear regression.

### Anchorage-independent cell growth assay

For anchorage-independent cell growth, 8000 cells of HCC827 and HCC827GR seeded onto soft agar. This assay was conducted as described previously [16]. The cells were incubated with various concentrations of ARu-B, Ru-B, GEF (1 μM), or SAV (2 nM) at 37°C in incubator. After two weeks, the colonies (more than 40 μm) were counted using a microscope and analysis software (IMT iSolution System; Vancouver, Canada).

### ADP-Glo kinase assay

EGFR, MET, AKT1, and AKT2 kinase activity were detected using an ADP-Glo Kinase Assay Kit (Promega, Madison, USA) according to the manufacturer’s protocols. In vitro kinase assay performed as described previously [17]. Inhibitors of the active kinase (EGFR, MET, AKT1, and AKT2) were added GEF, SAV, AZD5363, Ru-B, or ARu-B. The reaction was incubated in kinase detection buffer for 30 min and measured by a plate-reading luminometer (Berthold Technologies, Bad Wildbad, Germany).

### Molecular docking

The interaction between ARu-B and protein kinases EGFR or MET was predicted by molecular modeling using AutoDock Vina [18]. The structure files of EGFR and MET were downloaded from Protein Data Bank and the pdb id is 1M17 and 4XYF, respectively. The search grid was set to be either ATP-binding pocket or substrate binding site, in addition to the whole protein surface to see where the ligand is preferably located. Autodock simulation was accepted when the simulation produced 10 different modes, and the best modes were selected for molecular depiction.

### Western blotting

Proteins were extracted with Protein Extraction Solution (iNtRON Biotechnology, Seongnam, KR) containing with protease inhibitor. Concentration of proteins were measured using a BCA assay kit (Bio-Rad, CA, USA) and 30 μg of samples were separated by SDS-PAGE. The samples were electro-transferred to polyvinylidene difluoride (PVDF) membrane and blocked with 3–5% skim milk. The membrane was exposed with primary antibodies for overnight. After the membrane washed in PBS-Tween and then exposed HRP-secondary antibodies (Thermo Fisher Scientific) for 2 h. Target proteins were detected by ImageQuant™ LAS500 System (GE Healthcare, Uppsala, Sweden) and iBright™ CL1500 Imaging System (Thermo Fisher Scientific). The blot images were quantified by ImageJ software (NIH, Bethesda, MD), and the original full-length images are provided in the Supporting information (S1 File).

### Analysis of cell cycle

The NSCLC cells were incubated overnight and then treated with different concentration of ARu-B for 48 h. Collected cells were washed in PBS and then fixed in cold 70% Ethanol for 12 h. After washed cells were incubated with propidium iodide (PI) and observed with Muse™ Cell Analyzer (Merck Millipore, MA, USA).

### Measurement of reactive oxygen species (ROS)

Total ROS of cells was detected under a Muse™ Oxidative Stress Kit (Merck Millipore). The harvested cells with assay buffer were incubated with working solution for 30 min and observed using a Muse™ Cell Analyzer.

### Analysis of apoptosis

Early and lately apoptotic cells were measured using an Annexin V & Dead Cell Kit (Merck Millipore). Following the treatment, cells were collected with the culture media containing 5% fetal bovine serum. The samples were prepared as described previously [19]. The apoptotic cells were quantified by Muse™ Cell Analyzer.

### Mitochondrial membrane potential (MMP) assay

MMP was assessed by flow cytometry using Muse™ Mitopotential Kit (Merk Millipore). Cells treated with ARu-B as indicated were collected and stained as described previously [19]. Mitochondrial membrane potentials were quantified by Muse™ Cell Analyzer.

### MultiCaspase/7-AAD staining

The Muse™ MultiCaspase Kit (Merck Millipore) was used to measure the active caspase cells. Following the manufacturer’s instruction, 300 μL of cells were stained with 5 μL of Multi Caspase reagent for 30 min at 37°C. After adding 7-AAD working solution, samples were analyzed by Muse™ Cell Analyzer.

### Statistical methods

All data were expressed as mean±SD and analyzed using a KaleidaGraph 4.5 software (Synergy Software, PA, USA). One-way or two-way (ANOVA) following the Tukey’s post hoc test was used to analyze differences between more than two groups. Statistical significance was indicated by asterisks (*p < 0.05, **p < 0.01, and ***p < 0.001).

## Results

### Ru-B and ARu-B inhibit the proliferation of NSCLC cells

**Fig 2:**
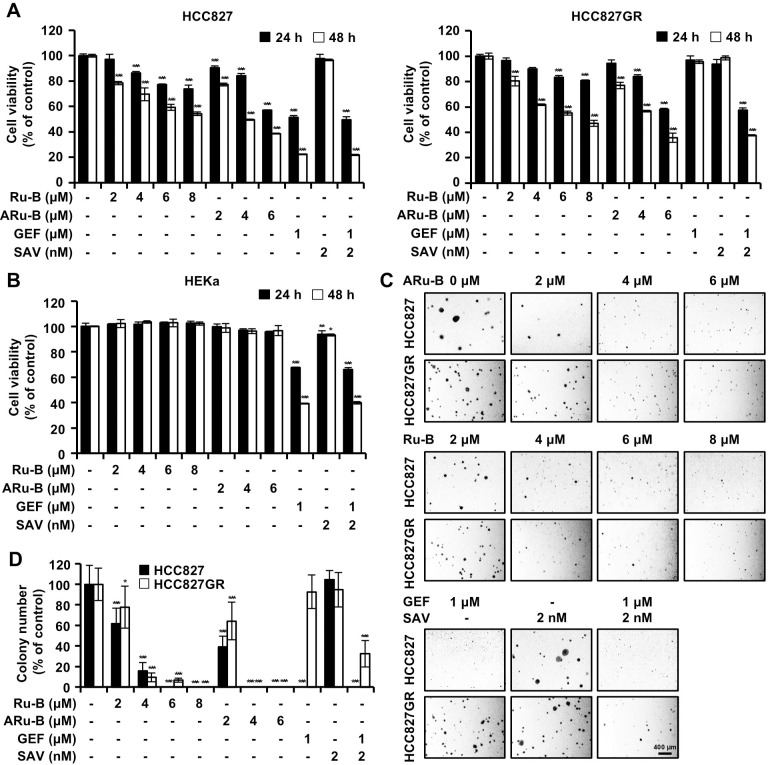
Growth inhibition of NSCLC cells by Ru-B and ARu-B. (A) Viability of NSCLC cells (HCC827 and HCC827GR) treated for 24 (black) and 48 h (white) with Ru-B (2, 4, 6, and 8 µM), ARu-B (2, 4, and 6 µM), gefitinib (GEF, 1 µM), and savolitinib (SAV, 2 nM) as monitored by the MTT assay. Data are shown as the mean ± SD (n = 3). (B) Cell viability of HEKa cells treated as in (A). (C) and (D) The soft agar assay was used to determine anchorage-independent colony growth in NSCLC cells (14 days of incubation). (C) Micrograph of the cells on day 14 and (D) colony number. *p < 0.05, **p < 0.01, and ***p < 0.001 compared to vehicle only. Scale bar, 400 µm.

We used the MTT cell viability assay to determine the effect of Ru-B and ARu-B on proliferation in HCC827 and HCC827GR cells (Fig 2A). In the MTT assay, the cell viability was 78.4%, 69.6%, 59.2%, and 54.1% in HCC827 cells, 80.4%, 61.8%, 55.2%, and 47.0% in HCC827GR cells after treatment with Ru-B (2, 4, 6, and 8 μM) for 48 h. After treatment with 2, 4, and 6 μM ARu-B for 48 h, the cell viability was 77.2%, 49.4%, and 38.6%, respectively, in HCC827 cells, and 76.9%, 56.7%, and 35.7% in HCC827GR cells. In HCC827 and HCC827GR cells, the IC_50_ values of Ru-B were 8.8 μM and 7.3 μM, respectively, at 48 h. The IC_50_ values of ARu-B in HCC827 and HCC827GR cells were 4.0 and 4.6 μM, respectively. The MTT assay results showed that HCC827 cells were sensitive to GEF (1 μM) but not to SAV (2 nM), a MET inhibitor. In HCC827GR cells, GEF (1 μM) treatment alone did not affect cell viability, whereas GEF combined with 2 nM SAV significantly decreased HCC827GR cell viability, as shown in Fig 2A. Additionally, treatment of HEKa cells with either ARu-B or Ru-B alone at 2, 4, 6, and 8 μM was not cytotoxic (Fig 2B). The IC_50_ values of ARu-B and Ru-B in HEKa cells were determined to be 23.5 μM and 37.7 μM, respectively (Table 1). In the colony formation assay, Ru-B (2, 4, 6, and 8 μM) decreased the relative number of HCC827 cell colonies to 61.7%, 15.9%, 0.0%, and 0.0%, respectively, relative to untreated cells and to 77.7%, 9.5%, 6.8%, and 0.0% in HCC827GR cells (Fig 2C and 2D). Colony formation inhibition was more apparent for ARu-B (2, 4, and 6 μM), where the relative number of HCC827 cell colonies was decreased to 39.3%, 0.0%, and 0.0%, respectively, and to 64.2%, 0.0%, and 0.0% in HCC827GR cells. Treatment with GEF alone decreased the number of HCC827 cell colonies but not those of HCC827 cells, which indicated GEF resistance in HCC827GR cells. However, co-treatment with both GEF and SAV significantly decreased the number of HCC827GR cell colonies.

**Table 1:** IC_50_ values of ARu-B and Ru-B in inhibiting the growth of NSCLC and HEKa cells (48 h, µM).

	HCC827	HCC827GR	HEKa
ARu-B	4.0	4.6	23.5
Ru-B	8.8	7.3	37.7

### Inhibition of EGFR and MET kinases by ARu-B

**Fig 3:**
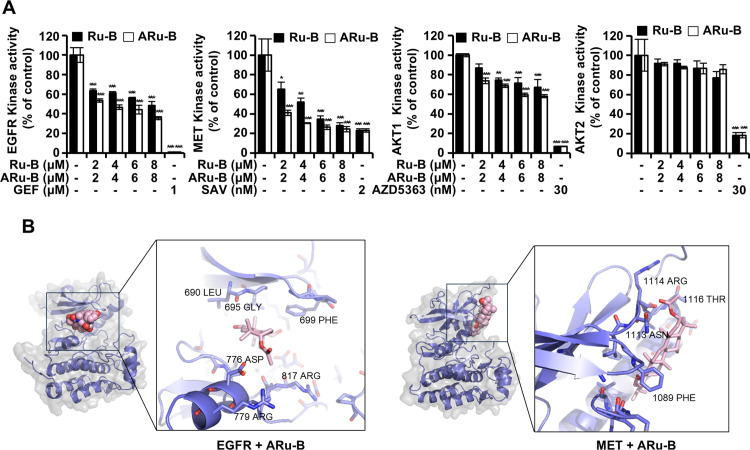
Inhibitory effect of Ru-B and ARu-B treatment on protein kinases. (A) *In vitro* ADP-Glo kinase activity assay for EGFR, MET, AKT1, and AKT2 to determine the inhibitory effects of Ru-B, ARu-B, gefitinib (GEF), savolitinib (SAV), or capivasertib (AZD5363). Data are shown as the mean ± SD (n = 3). *p < 0.05, **p < 0.01, and ***p < 0.001 compared with the control group. (B) Molecular modeling of ARu-B binding with EGFR and MET protein kinases. Bird’s eye view: Protein kinases are shown as surface and cartoon with ARu-B in spheres. Zoomed in: ARu-B (pink) and surrounding amino acids (purple).

An *in vitro* kinase assay was performed to determine if Ru-B and ARu-B modulated the activity of EGFR, MET, and AKT kinases. As shown in Fig 3A, the kinases were incubated with Ru-B or ARu-B. GEF (1 μM), SAV (2 nM), and AZD5363 (30 nM) were used as EGFR, MET, and AKT kinase inhibitors, respectively. Ru-B at 2, 4, 6, and 8 μM decreased the relative kinase activity of EGFR to 63.7%, 61.3%, 56.3%, and 48.5%, respectively, and that of MET kinase to 65.1%, 52.0%, 34.6%, and 28.0%. The inhibition was slightly greater for ARu-B (2, 4, 6, and 8 μM), and the relative kinase activity of EGFR dropped to 53.4%, 46.7%, 44.2%, and 35.7%, and that of MET kinase to 41.1%, 30.5%, 26.3%, and 24.4%. The IC_50_ values for EGFR were 7.6 μM and 3.0 μM for Ru-B and ARu-B, respectively. Similarly, the corresponding IC_50_ values for MET were 4.2 μM and 1.7 μM. AKT1 and AKT2 kinases were only moderately inhibited by Ru-B and ARu-B. The relative kinase activity of AKT1 and 67.1% and 58.0% respectively for Ru-B and ARu-B at 8 μM, and the corresponding values for AKT2 were 77.0% and 85.8%, indicating that AKT kinases were inhibited less compared to EGFR and MET kinases. Molecular docking simulation [18] placed the entire molecule of ARu-B in the ATP-binding pocket of EGFR kinase (Fig 3B), supporting the inhibition of EGFR by ARu-B and Ru-B. In the docking model, the 776 Asp residue of EGFR was near the acetyl group of the ARu-B, indicating a possible interaction between ARu-B and EGFR kinase. While the actual docking scores were comparable, −9.0 and −9.4 kcal/mol, the *in vitro* kinase assay and molecular docking results indicated that ARu-B was more potent in inhibiting EGFR and MET kinases than Ru-B. The docking simulations between ARu-B and MET kinase did not place the ARu-B molecule in the ATP-binding pocket but in the substrate-binding site.

### ARu-B inhibits signaling through EGFR/MET/AKT kinases

**Fig 4:**
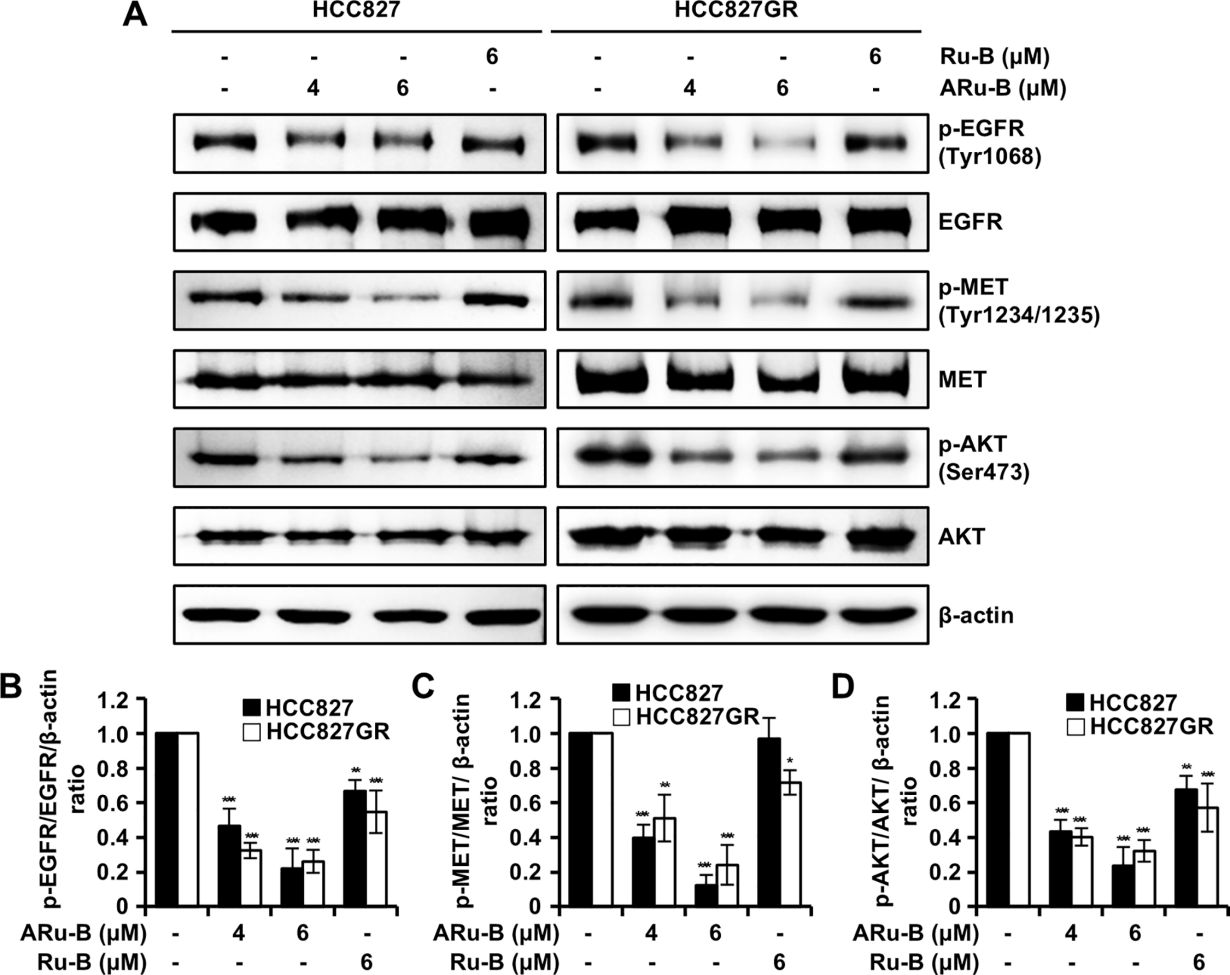
Inhibitory effects of Ru-B and ARu-B treatment on EGFR, MET, and AKT signaling. (A) HCC827 and HCC827GR non-small cell lung cancer (NSCLC) cells were treated with Ru-B (6 µM) or ARu-B (4 and 6 µM) for 48 h before western blotting to detect p-EGFR (Y1068), EGFR, p-MET (Y1234/1235), MET, p-AKT (S473), and AKT. β-actin was used as the loading control. (B)-(D) Histograms of protein expression analysis. Data are shown as the mean ± SD (n = 3). *p < 0.05, **p < 0.01, and ***p < 0.001 compared with the control group.

The level of phosphorylated proteins was monitored in HCC827 and HCC827GR cells treated with ARu-B (4 and 6 μM) or Ru-B (6 μM) by western blot analysis to examine if ARu-B modulated the EGFR/MET/AKT signaling axis. In both HCC827 and HCC827GR cells treated with 6 μM ARu-B, the level of phosphorylated EGFR, MET, and AKT decreased significantly compared to the untreated cells, with the total protein levels relatively unchanged (Fig 4A–D). Treatment with 6 μM Ru-B did not decrease the level of phosphorylated proteins as much as ARu-B. These results suggest that ARu-B, rather than Ru-B, suppresses signaling through EGFR/MET/AKT kinases.

### ARu-B induces cell cycle arrest at G0/G1 in NSCLC cells

**Fig 5:**
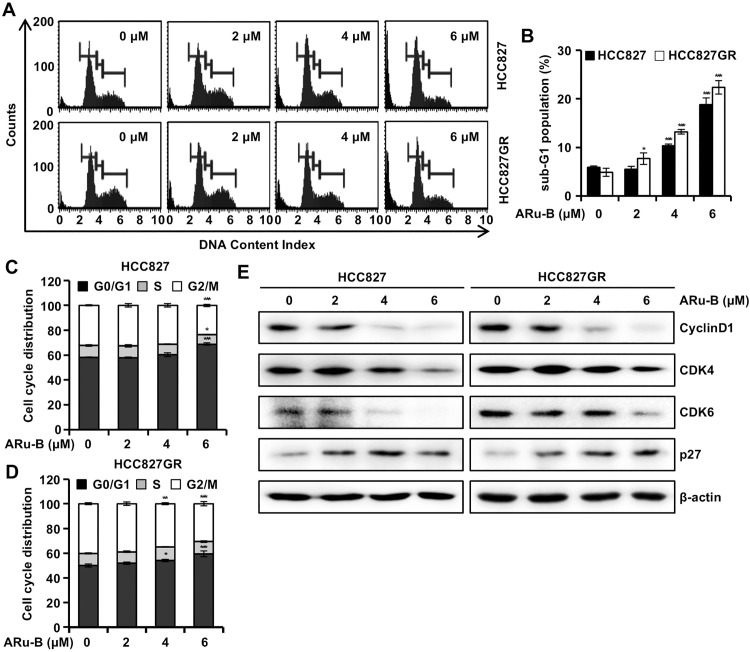
Cell cycle arrest by ARu-B. HCC827 and HCC827GR non-small cell lung cancer (NSCLC) cells were treated with ARu-B (0, 2, 4, and 6 µM) for 48 h before flow cytometric analysis of propidium iodide (PI) staining. (A) Flow cytometry plots. (B) Proportion of sub-G1 cells. (C, D) Cell cycle distribution. (E) Western blots of cyclin D1, CDK4, CDK6, and p27 proteins. β-actin was used as the loading control. *p < 0.05, **p < 0.01, and ***p < 0.001 compared with the control group.

The role of ARu-B in the cell cycle regulation of NSCLC cells was investigated using flow cytometry and propidium iodide (PI) staining. Treatment with 6 μM ARu-B increased the proportion of HCC827 cells in the sub-G1 phase from 5.9% to 18.8% and from 4.9% to 22.4% in HCC827GR cells (Fig 5A and 5B). In addition, the proportion of HCC827 cells in the G0/G1 phase increased from 58.2% to 68.8% and from 50.0% to 59.5% in HCC827GR cells (Fig 5C and 5D). Western blot analysis revealed that ARu-B treatment decreased the expression of cyclin D1, CDK4, and CDK6. However, ARu-B treatment significantly increased p27 levels in both HCC827 and HCC827GR cells. These results suggest that ARu-B treatment exerted antiproliferative activity by inducing cell cycle arrest at the G0/G1 phase in NSCLC cells.

### ARu-B induces an increase in cellular ROS levels in NSCLC cells

**Fig 6:**
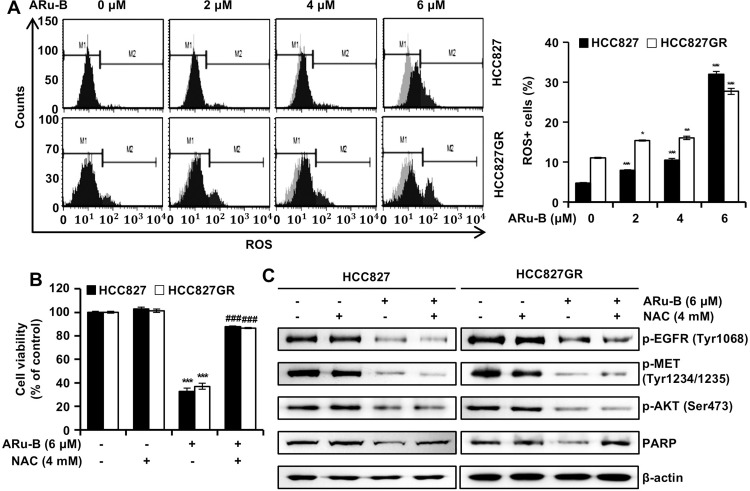
Induction of excessive ROS generation by ARu-B treatment. HCC827 and HCC827GR non-small cell lung cancer (NSCLC) cells were treated with ARu-B (0, 2, 4, and 6 µM) for 48 h before flow cytometric analysis using a Muse™ Oxidative Stress Kit. (A) Flow cytometry plots (left) showing ROS levels detected using Muse™ Oxidative Stress Kit, which identifies ROS-positive cells based on DCF fluorescence intensity. M1 represents ROS-negative, and M2 indicates ROS-positive cell populations. Quantification (right) represents the percentage of M2-gated ROS-positive cells. (B, C) NSCLC cells pretreated with N-acetyl-L-cysteine (NAC, 4 mM) or vehicle were incubated with ARu-B for 48 h. (B) Cell viability was measured using the MTT assay. (C) Western blot analysis of phosphoproteins and proteins: p-EGFR, p-MET, p-AKT and poly (ADP-ribose) polymerase (PARP). β-actin was used as the loading control. *p < 0.05, **p < 0.01 and ***p < 0.001 compared with the control group. ^###^p < 0.001 compared with ARu-B treatment.

A Muse cell analyzer was used to determine the level of cellular ROS in NSCLC cells treated with ARu-B. In HCC827 cells treated with ARu-B (2, 4 and 6 μM), the level of ROS increased from 4.7% to 7.9%, 10.5%, and 32.0%, respectively, showing a concentration-dependent response (Fig 6A). Likewise, the level of ROS in HCC827GR cells increased from 11.0% to 15.4%, 16.0%, and 27.7%, respectively, with the corresponding concentrations of ARu-B. To determine if ROS increases were crucial to the antiproliferative effect of ARu-B, HCC827 cells were pretreated with the ROS scavenger N-acetylcysteine (NAC; 4 mM) for 3 h, and the cell viability was assessed using the MTT assay (Fig 6B). Indeed, NAC pretreatment partially rescued the ARu-B-induced cytotoxicity, confirming the involvement of ROS in cell death. The viability of HCC827 and HCC827GR cells, which dropped to 34.8% and 36.4%, respectively, rebounded to 87.8% and 86.6% with NAC pretreatment. Even though cell viability recovered, decreases in the phosphorylated kinases EGFR, MET, and AKT were not mitigated (Fig 6C). However, NAC pretreatment prevented the cleavage of caspase-3 and PARP, indicating ROS plays a role upstream of caspase activation.

### ARu-B induces the apoptosis of NSCLC cells by activating caspases

**Fig 7:**
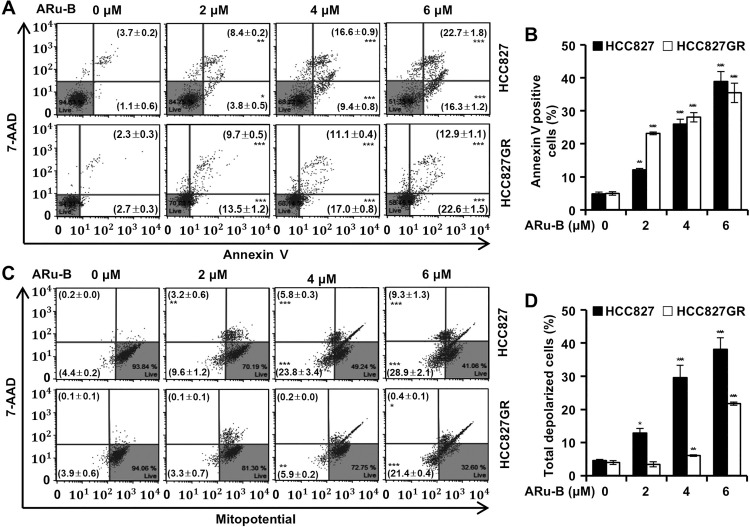
ARu-B-induced apoptosis mediated by mitochondrial membrane depolarization. HCC827 and HCC827GR NSCLC cells were treated with ARu-B (0, 2, 4, and 6 µM) for 48 h before flow cytometric analysis with annexin V/7-AAD double staining and Mitopotential/7-AAD double staining assay. (A) Annexin V/7-AAD double staining assay. Early and late apoptotic cells were distinguished using Annexin V-FITC/7-AAD staining; early apoptotic cells are Annexin V⁺/7-AAD⁻, and late apoptotic cells are Annexin V⁺/7-AAD⁺. (B) Proportion of apoptotic cells. (C) Mitopotential/7-AAD double staining assay. Left side: cells with depolarized mitochondrial membrane. (D) Proportion of depolarized cells. *p < 0.05,**p < 0.01, and ***p < 0.001 compared with the control group.

**Fig 8:**
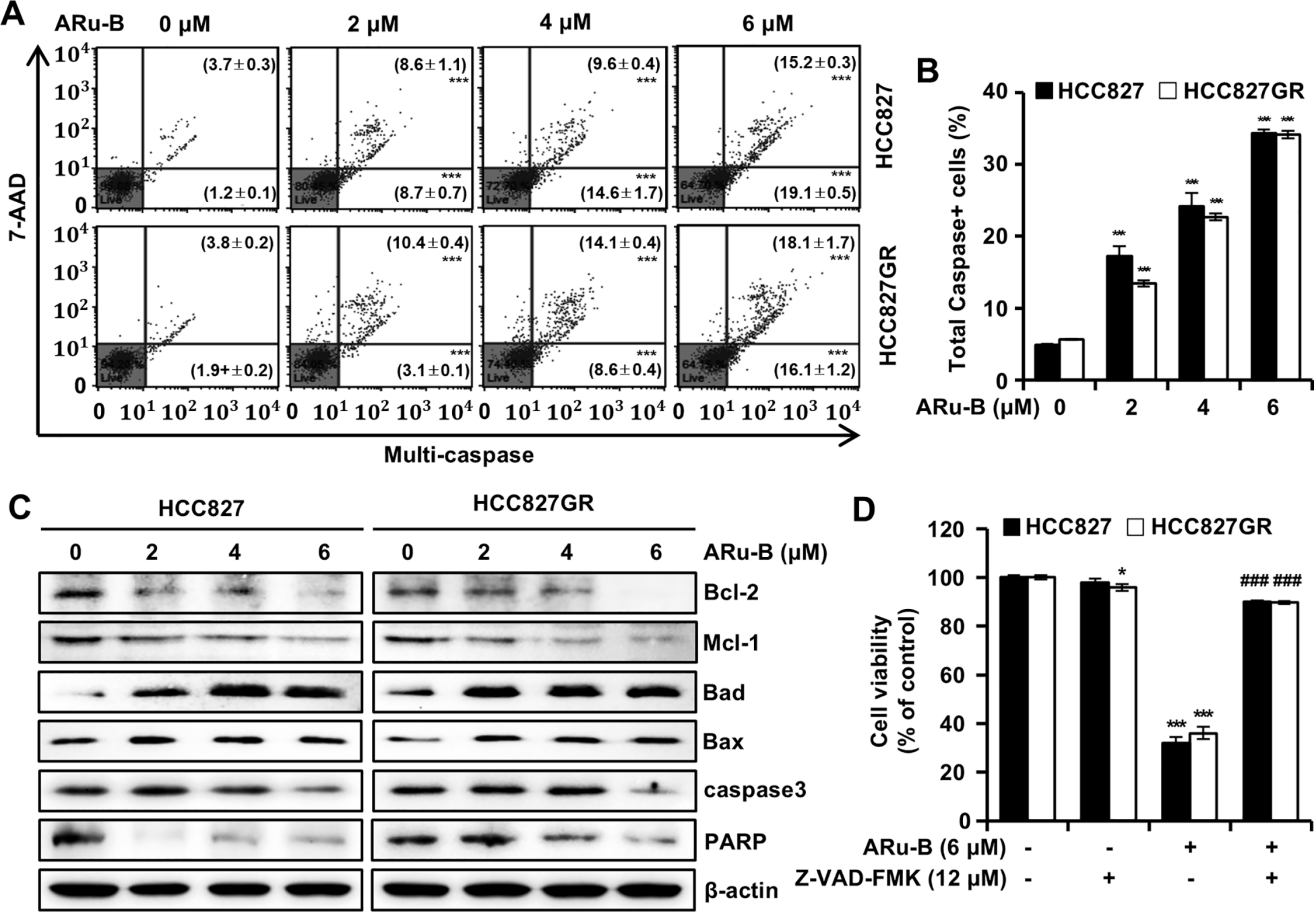
ARu-B-induced apoptosis mediated by caspase activation. HCC827 and HCC827GR NSCLC cells were treated with ARu-B (0, 2, 4, and 6 µM) for 48 h before flow cytometric analysis using a Muse™ Multi-Caspase Kit. (A) Multi-Caspase assay. (B) Proportion of cells with caspase activation. (C) Western blot analysis of Bcl-2, Mcl-1, Bad, Bax, caspase 3, and PARP. β-actin was used as the control. (D) NSCLC cells pretreated with Z-VAD-FMK (12 µM) or vehicle for 3 h were incubated with ARu-B for 48 h. Cell viability was measured using the MTT assay. *p < 0.05 and ***p < 0.001 compared with the control group. ###p < 0.001 compared with the ARu-B treatment group.

Flow cytometric analysis was performed to examine the proportion of NSCLC cells treated with ARu-B (2, 4, and 6 μM) undergoing apoptosis. The analysis was performed using annexin V/7-AAD double-staining with a MUSE^TM^ analyzer (Fig 7A and 7B). After 48 h of treatment with ARu-B (0, 2, 4, and 6 μM), the proportion of HCC827 cells in the early apoptotic phase (annexin V+/7-AAD-) increased from the background level of 1.1% to 3.8%, 9.4%, and 16.3%, respectively, and from 2.7% to 13.5%, 17.0%, and 22.6% in HCC827GR cells. Moreover, the ratio of HCC827 cells in the late apoptotic phase (annexin V+/7-AAD+) increased from 3.7% to 8.4%, 16.6%, and 22.7%, and from 2.3% to 9.7%, 11.1%, and 12.9% in HCC827GR cells. The induction of apoptosis was accompanied by mitochondrial membrane depolarization (Fig 7C and 7D). In addition, flow cytometric analysis with a Muse^TM^ MultiCaspase Kit revealed the activation of caspases in HCC827 cells treated with ARu-B (Fig 8A). The percentage of HCC827 cells with multi-caspase activity was observed to increase with ARu-B treatment (2, 4, and 6 μM) from 4.8% to 17.3%, 24.2%, and 34.3%, respectively, and from 5.7% to 13.5%, 22.7%, and 34.2% in HCC827GR cells (Fig 8A and 8B). Western blot analysis indicated that the balance in Bcl-2 family proteins was perturbed in NSCLC cells treated with ARu-B, as well as the decrease in the level of full-length caspase 3 and PARP (Fig 8C). NSCLC cells were pretreated with Z-VAD-FMK (multi-caspase inhibitor, 12 μM) for 3 h before ARu-B treatment to determine if caspase activation was crucial for the antiproliferative effect of ARu-B, and cell viability was determined using the MTT cell viability assay (Fig 8D). The viability of HCC827 and HCC827GR cells, which dropped to 32.0% and 36.0% respectively, with ARu-B treatment, was 90.0% and 89.6% with Z-VAD-FMK pretreatment in addition to ARu-B.

## Discussion

Triterpenoids, comprising 30 carbon atoms from biosynthesis, are one of the largest groups of natural products with various pharmacological activities [20]. Among them, pentacyclic triterpenoid compounds have been shown to have biological activities with the potential to treat multiple diseases, such as cardiovascular disease [21], metabolic syndromes [22], and cancer [23]. We previously identified a series of arborinane-type triterpenoids from the genus *Rubia* [24] and showed that Ru-B induced necroptosis in a colorectal cancer model [13]. In the present study, ARu-B, an acetylated form of Ru-B, exhibited superior anticancer activity in NSCLC cells compared to Ru-B. To our knowledge, this is the first report on the anticancer activity of ARu-B in a GEF-sensitive and -resistant NSCLC model.

ARu-B and Ru-B treatments showed antiproliferative activity in both GEF-sensitive and -resistant cells (Fig 2A). Even though the antiproliferative activity was not as potent as that of GEF alone (1 μM) or SAV alone (2 nM), it was time- and concentration-dependent, and GEF-resistance did not affect the antiproliferative activity of either ARu-B and Ru-B, implying that these compounds may serve as chemotherapeutic agents for treating NSCLC. HEKa cell viability was not affected under the same conditions. The selectivity index of ARu-B was 5.9, based on a comparison of IC₅₀ values between HCC827 and HEKa cells, indicating a high degree of selectivity toward cancer cells in exerting cytotoxicity. ARu-B may offer a therapeutic advantage over conventional EGFR or MET inhibitors which often exhibit broader toxicity profiles affecting normal epithelial cells. However, we cannot exclude the possibility of off-target effects, as ARu-B may affect other cellular components. EGFR, MET, and AKT signaling is important for cancer cell survival [25]. The in vitro kinase assay in the present study showed that EGFR, MET, and AKT1 were inhibited by ARu-B and Ru-B (Fig 3A). The molecular docking model suggested that ARu-B preferentially bound to EGFR rather than Ru-B with its acetyl group with 776 Asp, which is in the ATP-binding pocket (Fig 3B) [26]. ARu-B was placed in the substrate-binding site rather than the ATP-binding site when molecular docking was performed between ARu-B and MET kinase. While not as potent as other kinase inhibitors targeting MET kinase, inhibition was clearly observed in the *in vitro* kinase assay, suggesting that ARu-B might have multiple modes of binding to different kinase molecules. However, the question of whether and how ARu-B inhibits these kinases must be clarified. The acetyl group in ARu-B differentiates it from Ru-B in at least two distinct ways. First, the acetyl group could participate in interactions between ARu-B and various kinases, as suggested by the molecular docking study. Second, the hydrophobicity introduced by masking the hydroxyl group with an acetyl group could enhance permeability through the cell membrane, thereby increasing the intracellular availability of ARu-B in target cancer cells. Either way, ARu-B appears to preferentially inhibit the EGFR, MET, and AKT signaling pathways, as evidenced by decreases in the levels of phosphorylated kinases (Fig 4).

Cell cycle regulation is one of the mechanisms by which anticancer therapeutics exert cytotoxicity. G0/G1 arrest has been reported with first-generation EGFR inhibitors like gefitinib, indicating ARu-B may mimic aspects of known therapeutic responses [27]. In this study, ARu-B treatment induced cell accumulation in the G0/G1 phase (Fig 5C and 5D). This was accompanied by a reduction in proteins associated with cell cycle progression, including cyclin D1, CDK4, and CDK6, and an increase in p27, a CDK inhibitor (Fig 5E). These results suggest that the antiproliferative activity of ARu-B is partially attributable to cell cycle arrest at the G0/G1 phase.

Increases in cellular ROS levels can be cytotoxic in cancer cells [28]. ARu-B treatment induced the production of excessive amounts of ROS (Fig 6A). Pretreatment with 4 mM NAC prevented the cytotoxicity of ARu-B (Fig 6B), indicating that the production of extra ROS was crucial to the cytotoxicity. However, NAC pretreatment did not mitigate the decrease in phosphorylated EGFR, MET, and AKT, implying that the suppression of these kinases preceded ROS generation in ARu-B-induced apoptosis (Fig 6C).

ARu-B treatment increased the proportion of apoptotic cells (Fig 7A and 7B), cells with depolarized mitochondrial membrane (Fig 7C and 7D), and cells with activated caspases (Fig 8A and 8B). We speculate that ARu-B initiates the intrinsic apoptotic pathway, as the balance in the Bcl-2 family proteins was perturbed. Furthermore, ARu-B treatment resulted in the activation of caspase-3, as evidenced by decreases in the level of full-length caspase-3 and PARP proteins monitored by western blotting (Fig 8C). Z-VAD-FMK pretreatment prevented the ARu-B-induced cytotoxicity (Fig 8D). These results suggest that the ARu-B induced the apoptosis of NSCLC cells by activating caspases.

## Conclusions

**Fig 9:**
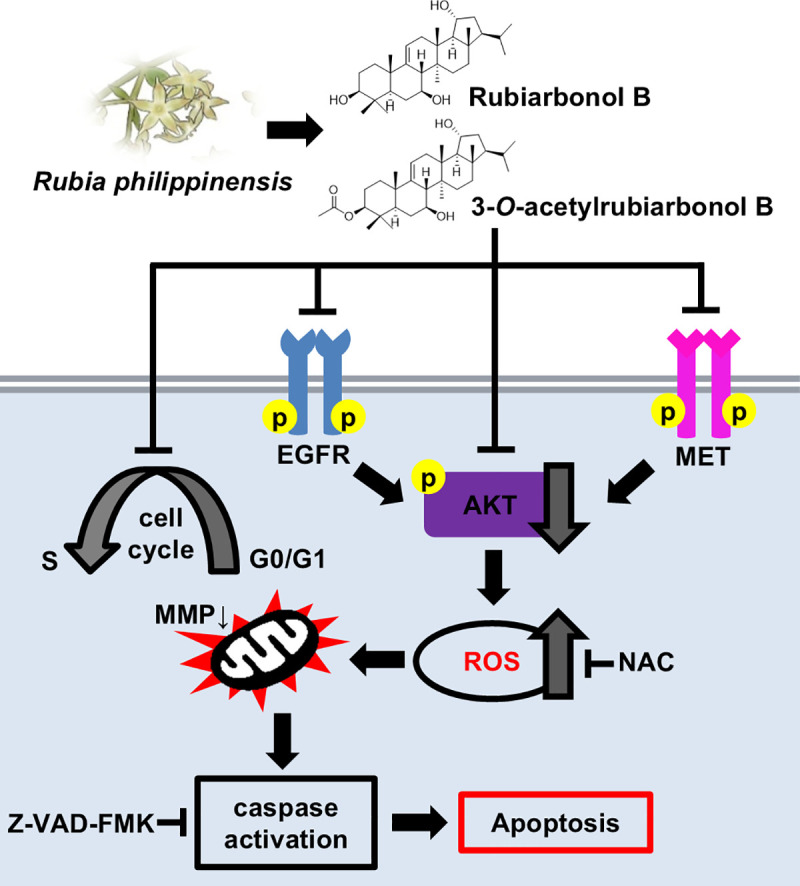
Proposed mechanism of apoptosis induced by ARu-B and Ru-B. ARu-B and Ru-B, isolated from *Rubia philippinensis*, induce apoptosis in non-small cell lung cancer (NSCLC) cells by targeting EGFR, MET, and AKT signaling pathways.

We demonstrated that ARu-B treatment inhibited the growth of NSCLC HCC827 cells, regardless of GEF resistance. The *in vitro* kinase assays revealed that EGFR, MET, and AKT1 were inhibited by ARu-B, which correlated with the cytotoxic effects observed in the cell culture model. Additionally, ARu-B induced cell cycle arrest, excessive ROS generation, and caspase activation (Fig 9). In addition to our results, in vivo studies such as xenograft models would help validate the anticancer effects of ARu-B. Further studies would elucidate the molecular targets of ARu-B, which could contribute to the development of improved treatments for lung cancer.

## Supporting information

S1 Fig ^1^H (A) and ^13^C NMR (B) spectroscopic data of rubiarbonol B (300 MHz, pyridine-*d*_5_). (TIF)

S1 Table ^1^H NMR (600 MHz) and ^13^C NMR (150 MHz) spectroscopic data of 3-*O*-acetylrubiarbonol B (in CDCl_3_). (PDF)

S1 File Raw images. (PDF)

S2 File Raw data set. (XLSX)
