# Supplementary figures and images for: 3-O-acetylrubiarbonol B preferentially targets EGFR and MET over rubiarbonol B to inhibit NSCLC cell growth
Source: PLoS One. 2025 Sep 8;20(9):e0329706. doi: 10.1371/journal.pone.0329706 (PMC12416685; doi:10.1371/journal.pone.0329706)

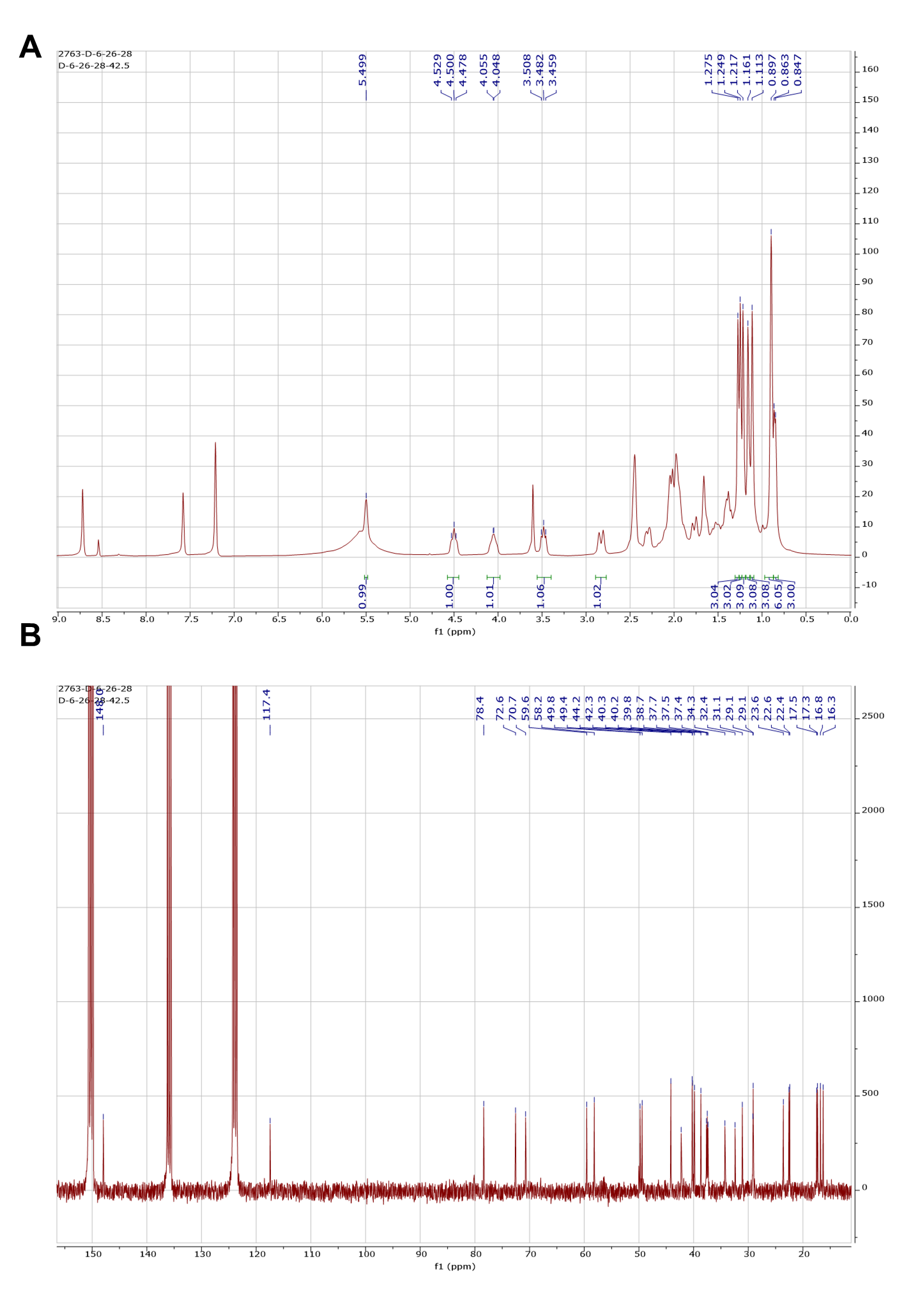

Supplement: S1 Fig — (TIF) [file pone.0329706.s001.tif]
