# Supplementary material for: 3-O-acetylrubiarbonol B preferentially targets EGFR and MET over rubiarbonol B to inhibit NSCLC cell growth
Source: PLoS One. 2025 Sep 8;20(9):e0329706. doi: 10.1371/journal.pone.0329706 (PMC12416685; doi:10.1371/journal.pone.0329706)
Supplement: S1 Table — (PDF) [file pone.0329706.s002.pdf]

S1 Table. ^{1}H NMR (600 MHz) and ^{13}C NMR (150 MHz) spectroscopic data of 3-*O*-acetylrubiarbonol B (in $CDCl_3$).

position	δ_H (*J* in Hz)	δ_C
1	1.75, ov.; 1.50, m	36.2
2	1.78, ov.; 1.67, ov.	24.3
3	4.47, dd (12.0, 4.2)	80.7
4		37.8
5	0.99, m	48.2
6	1.91, dd (12.3,5.4); 1.60, q (12.3)	32.9
7	3.76, m	72.1
8	2.07, br d (8.8)	49.1
9		146.1
10		39.3
11	5.32, br d (6.0)	117.3
12	1.76, ov; 1.61, ov	36.6
13		37.8
14		39.6
15	1.99, m; 1.62, d (12.3)	32.1
16	1.98, dd (16.0, 3.4); 1.78, dd (16.0, 5.6)	37.0
17		44.0
18	1.62, d (9.6)	59.5
19	4.21, td (9.6, 3.6)	71.5
20	1.86, m; 1.69, m	41.0
21	1.31, q (9.8)	57.4
22	1.43, m	30.5
23	0.87, s	28.1
24	0.89, s	16.7
25	1.08, s	21.9
26	0.97, s	16.9
27	0.91, s	16.8
28	0.80, s	15.7
29	0.88, d (6.6)	22.1
30	0.83, d (6.6)	23.0
OAc-3	2.05, s	171.0
		21.4

Ov.: overlapped resonances
