# Supplementary material for: 3-O-acetylrubiarbonol B preferentially targets EGFR and MET over rubiarbonol B to inhibit NSCLC cell growth
Source: PLoS One. 2025 Sep 8;20(9):e0329706. doi: 10.1371/journal.pone.0329706 (PMC12416685; doi:10.1371/journal.pone.0329706)

Original image for figure 4 – Repeat 1

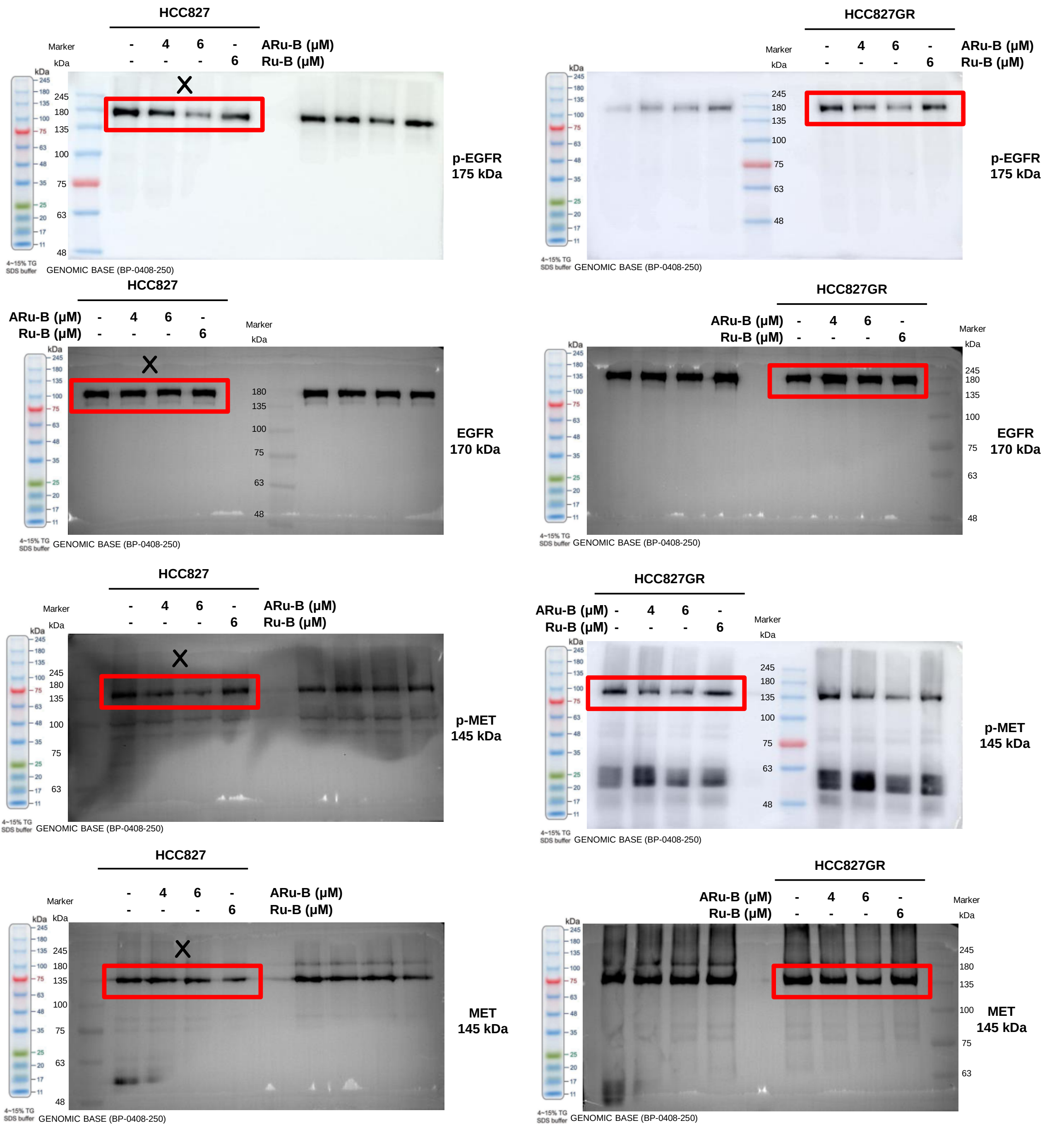

Original image for figure 4 – Repeat 1

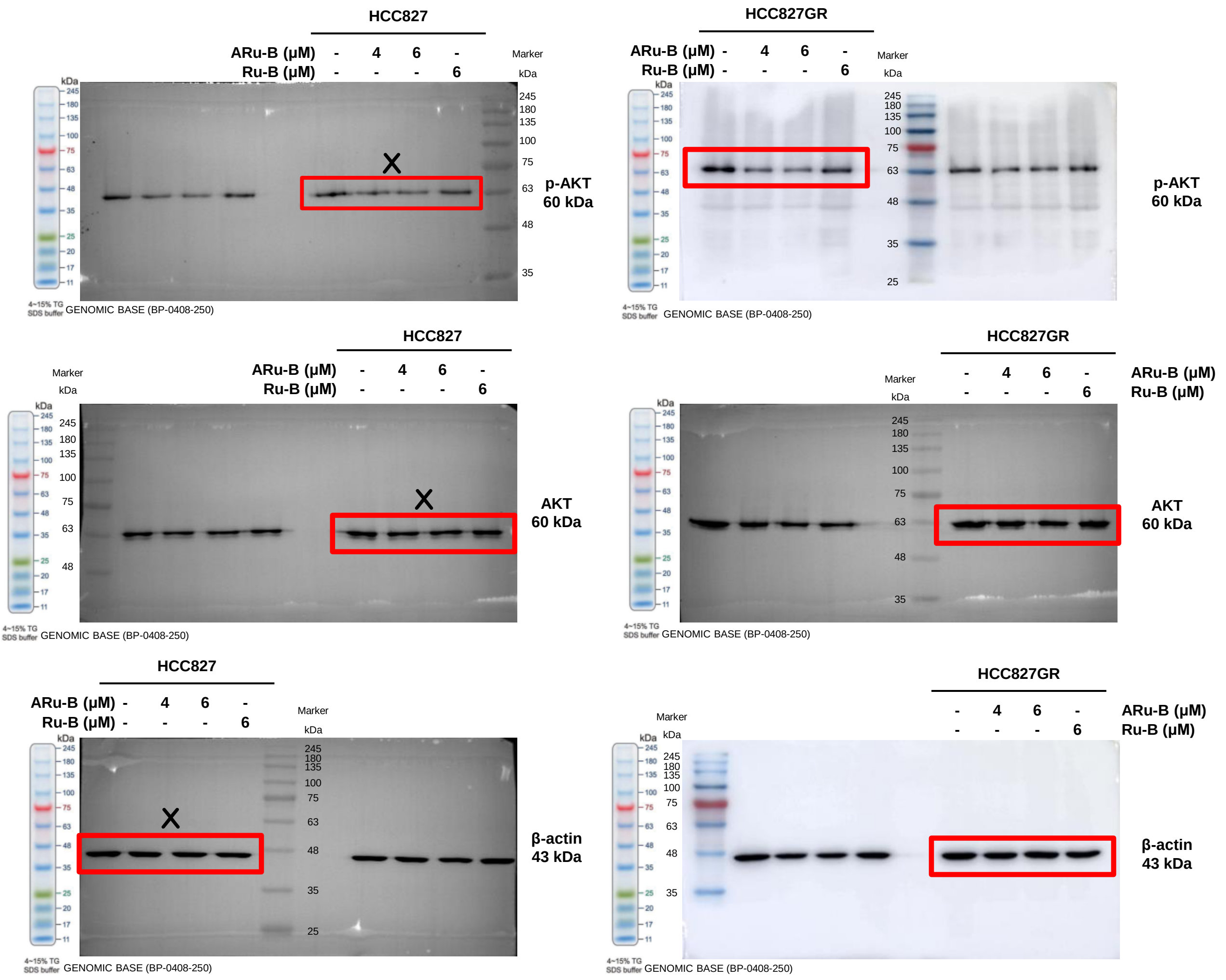

Original image for figure 4 – Repeat 2

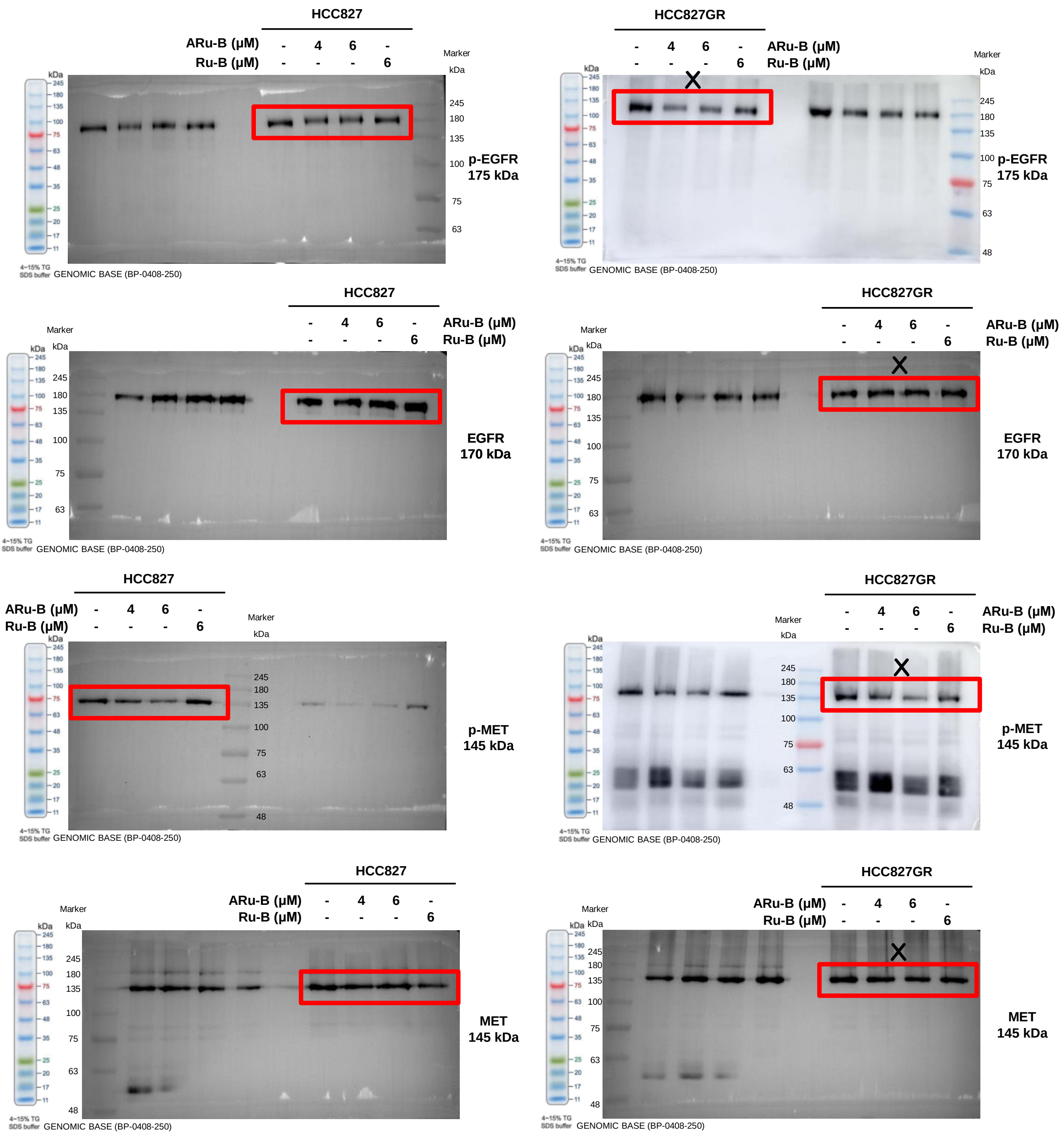

Original image for figure 4 – Repeat 2

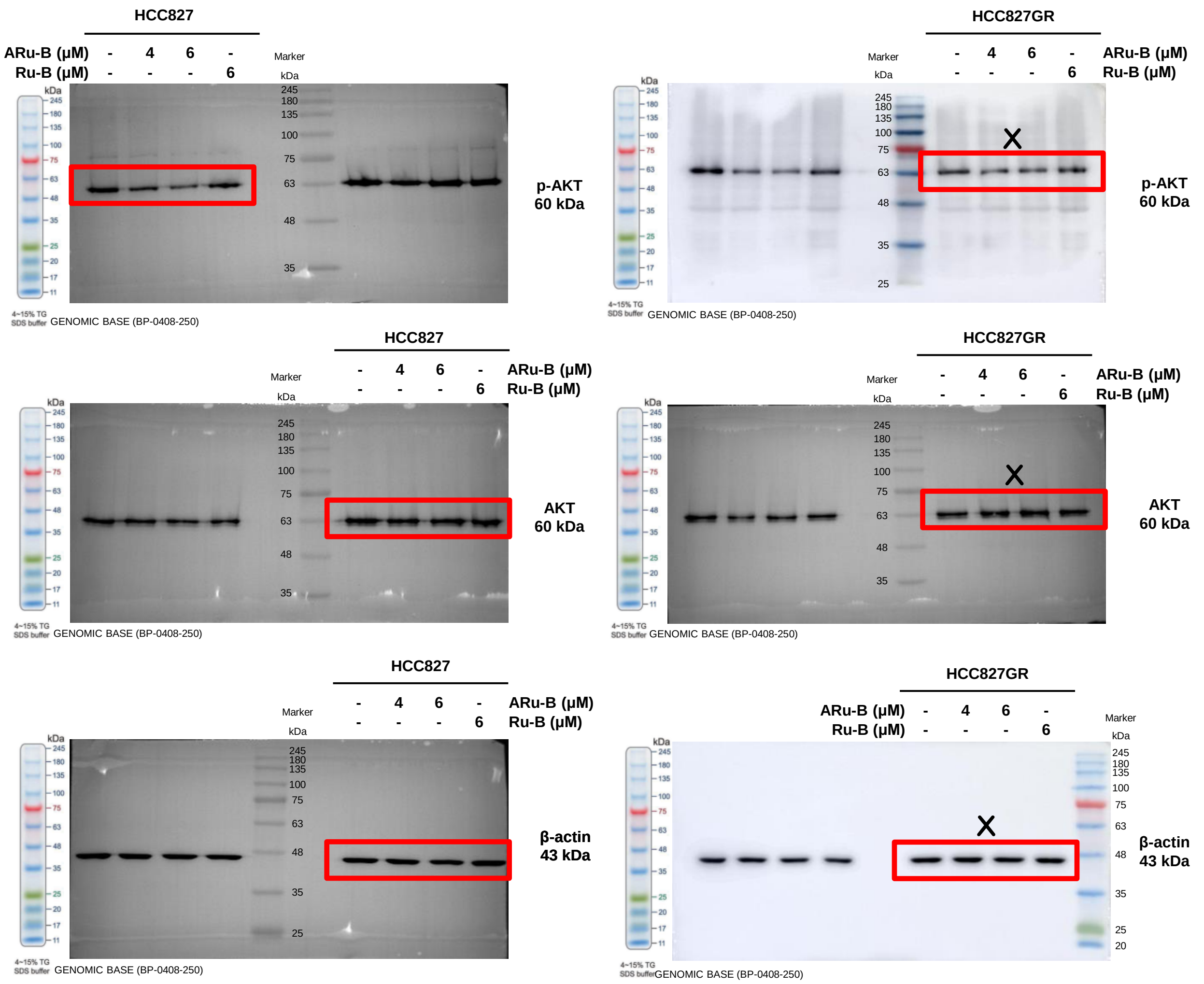

Original image for figure 4 – Repeat 3

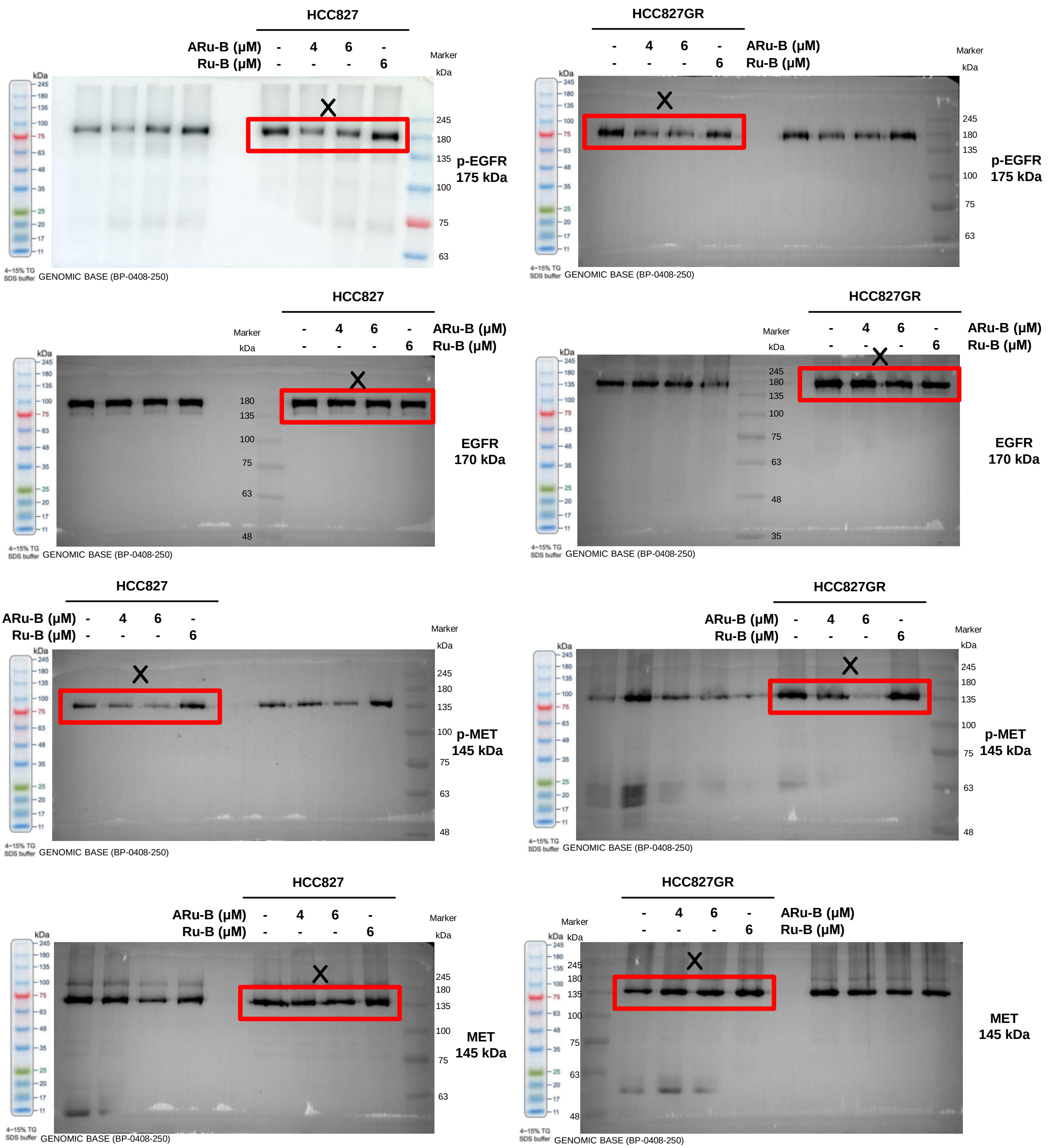

Original image for figure 4 – Repeat 3

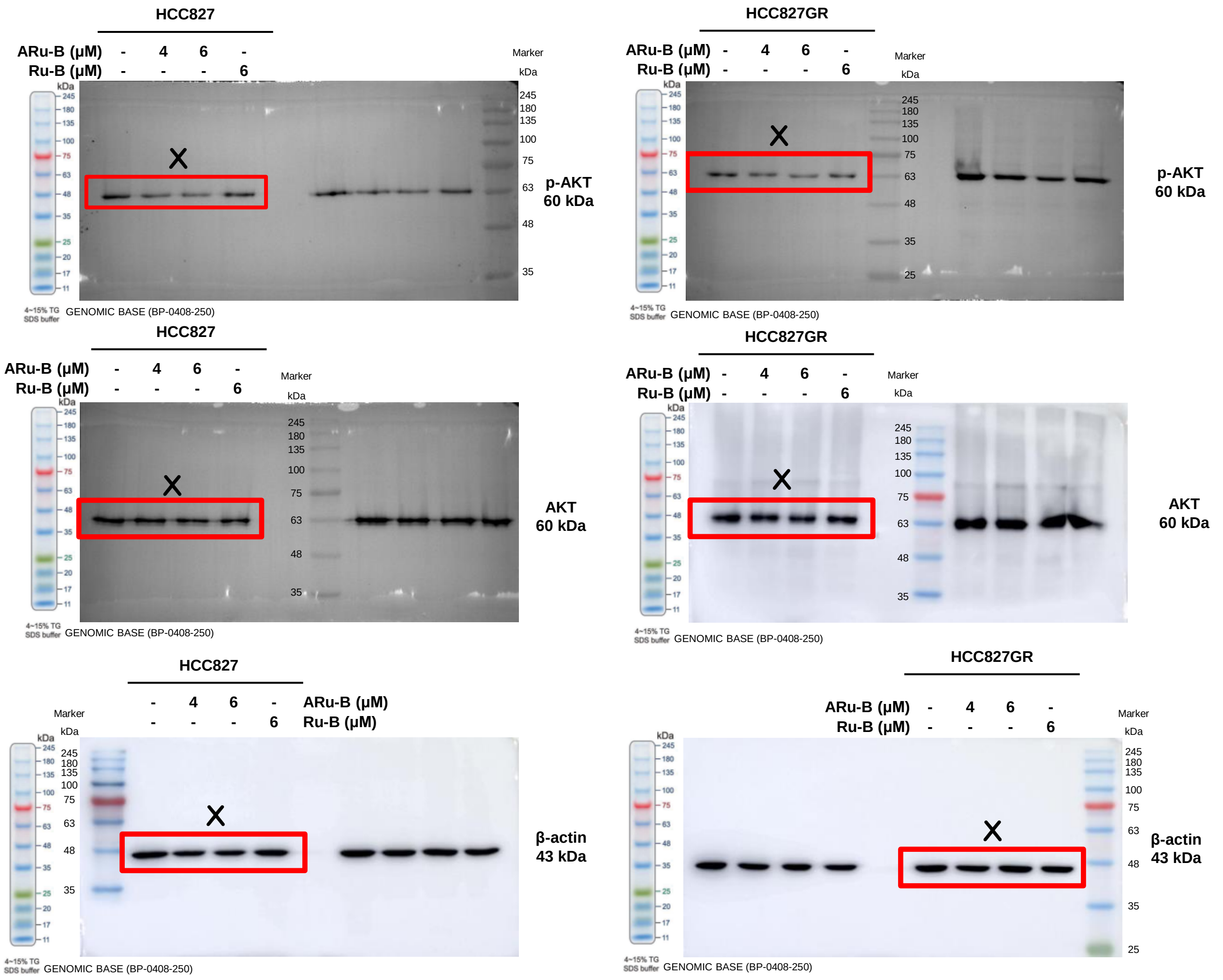

Original image for figure 5E

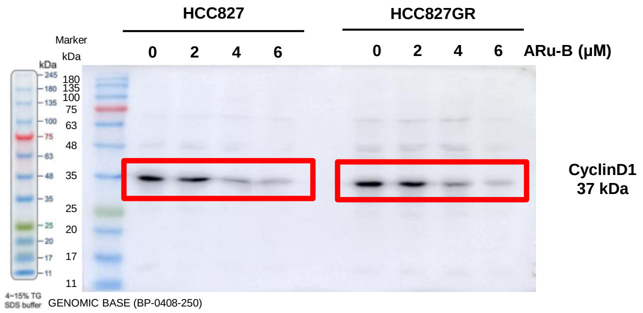

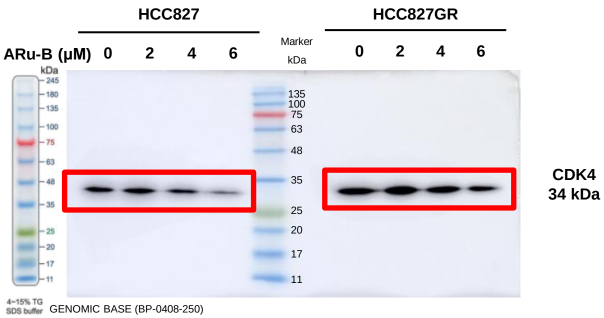

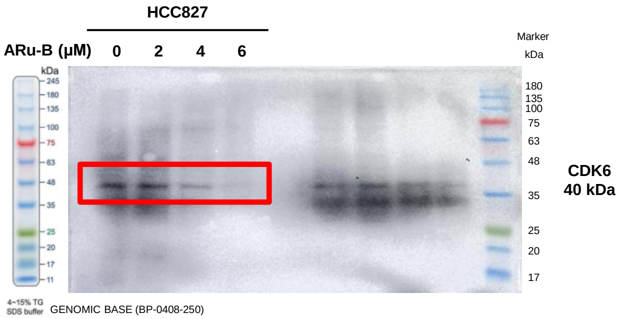

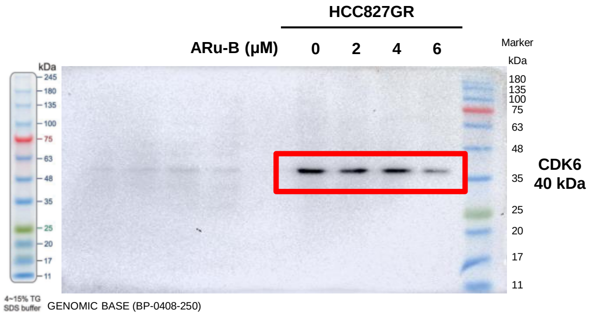

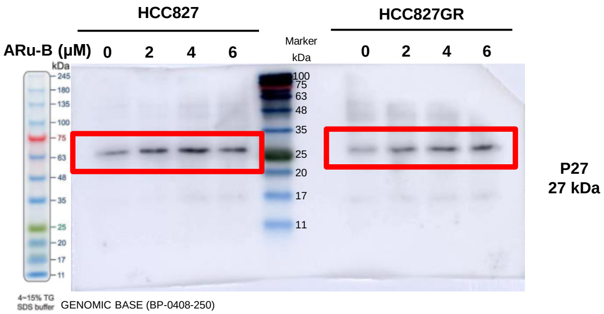

Original image for figure 5E

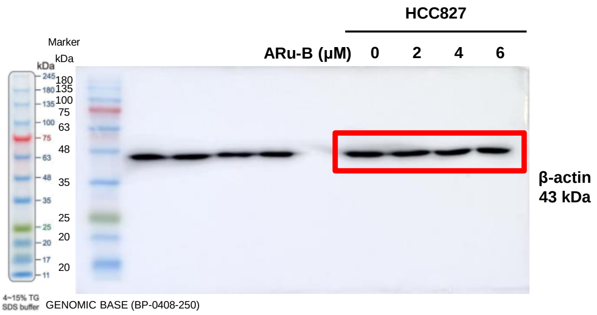

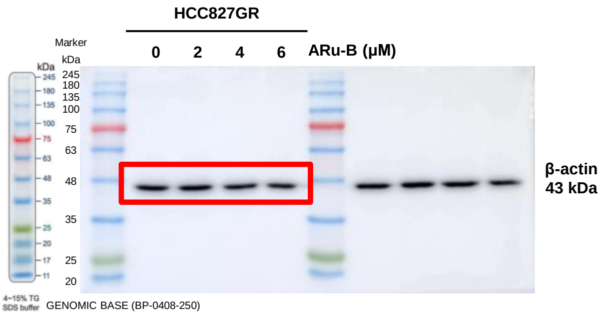

Original image for figure 6C

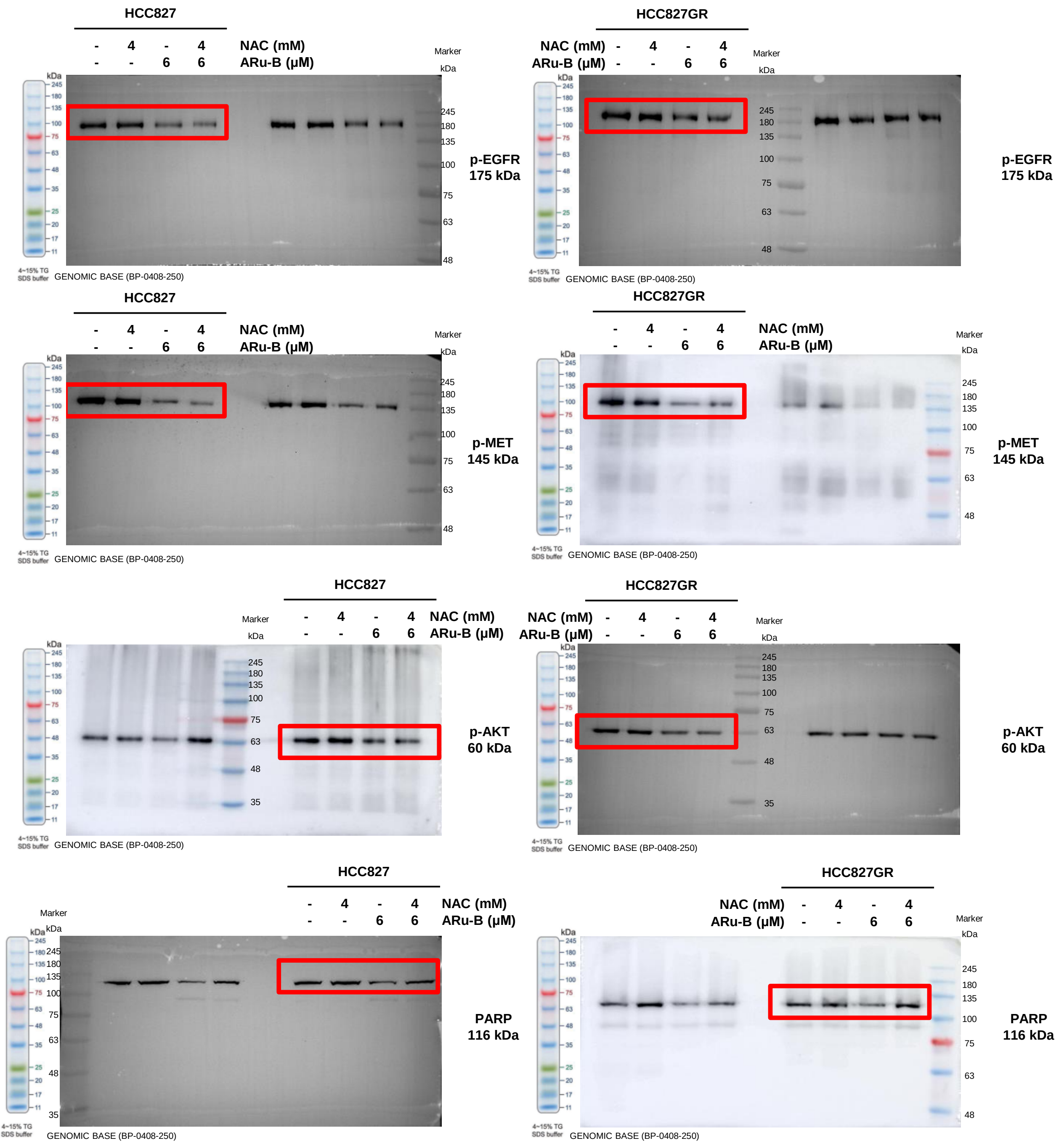

Original image for figure 6C

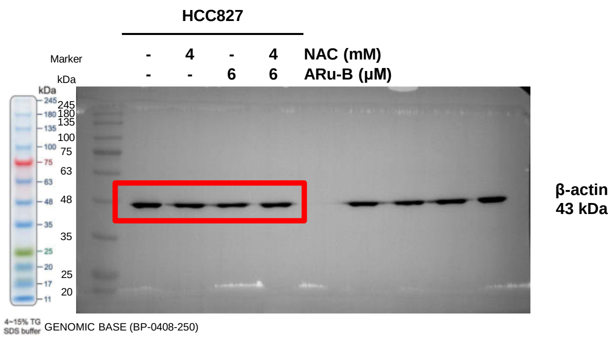

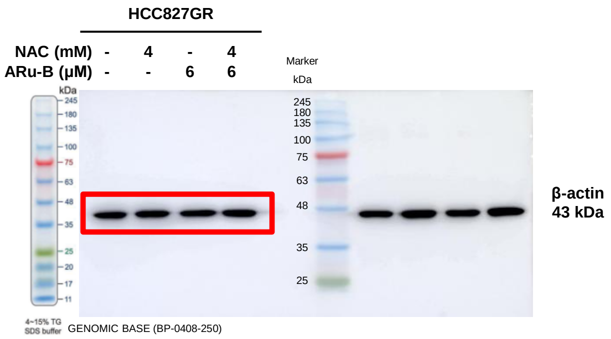

Original image for figure 8C

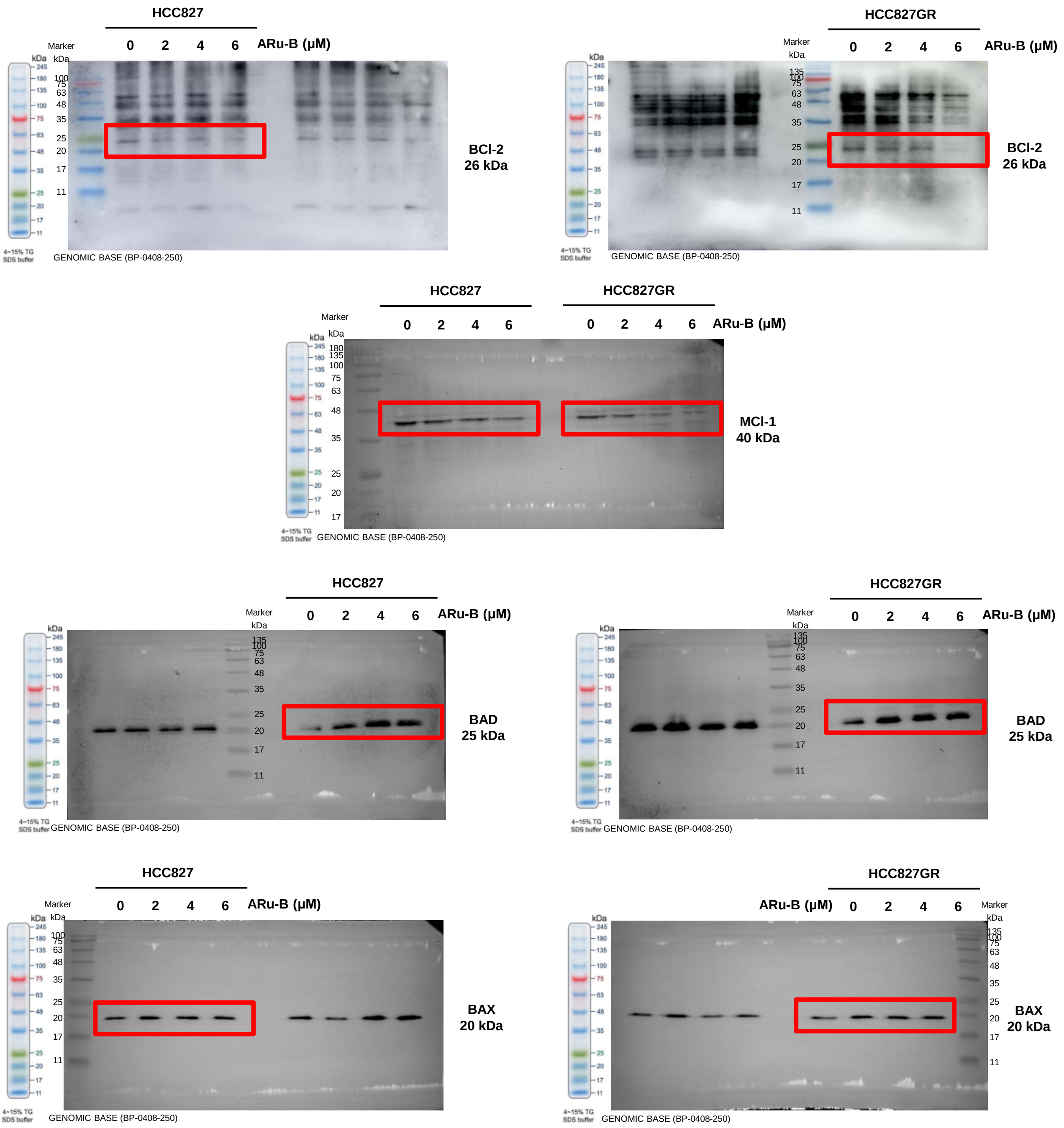

Original image for figure 8C

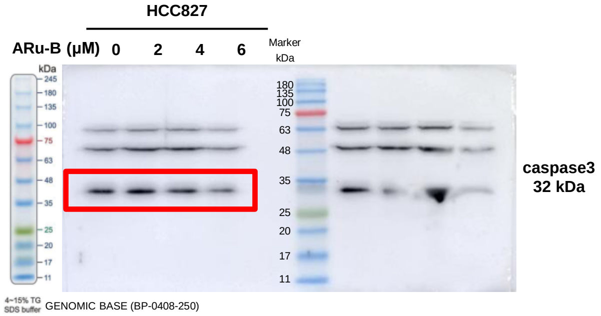

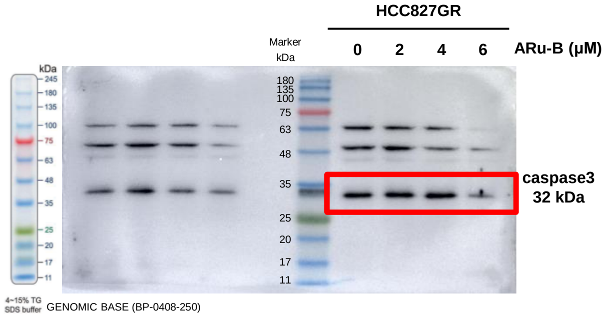

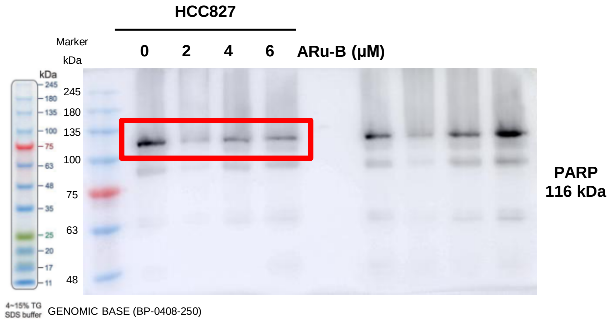

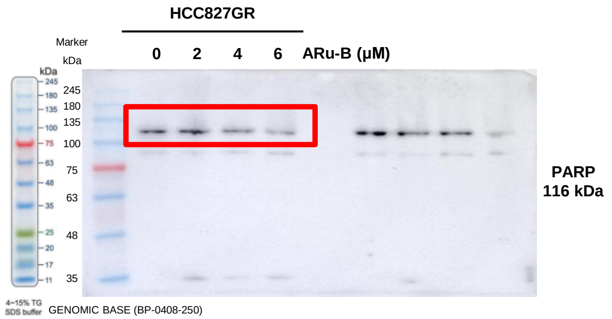

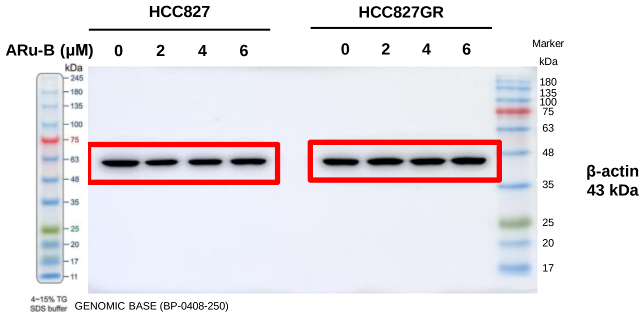

Supplement: S1 File — (PDF) [file pone.0329706.s003.pdf]
